# High N-Cadherin Protein Expression in Ovarian Cancer Predicts Poor Survival and Triggers Cell Invasion

**DOI:** 10.3389/fonc.2022.870820

**Published:** 2022-04-28

**Authors:** Mourad Assidi

**Affiliations:** ^1^ Center of Excellence in Genomic Medicine Research, King Abdulaziz University, Jeddah, Saudi Arabia; ^2^ Medical Laboratory Department, Faculty of Applied Medical Sciences, King Abdulaziz University, Jeddah, Saudi Arabia

**Keywords:** N-Cadherin, ovarian cancer, immunohistochemistry, prognosis, EMT, tissue microarray, survival

## Abstract

Ovarian cancer (OC) is among the most lethal cancer among all gynaecological malignancies. Since most OC patients are diagnosed only at advanced stages mainly because of their imperceptible/nonspecific symptoms, survival rates are low. Therefore, more molecular biomarkers are needed to achieve more effective molecular stratification for better prognostic and theranostic outcomes. The cadherin family, particularly N-cadherin (N-CAD; also known as CDH2), is critical for cell-cell adhesion and epithelial- mesenchymal transition (EMT) of cancer. N-CAD protein has also been shown to be overexpressed in many advanced carcinomas. The aim of this study was to investigate the expression patterns of N-CAD protein, determine their correlations with the clinicopathological features of OC patients, and evaluate its prognostic value and involvement in EMT and metastasis. Protein expression of N-CAD was studied in 117 formalin-fixed and paraffin-embedded (FFPE) blocks from patients diagnosed with OC using Tissue Microarray and immunohistochemistry techniques. The N-CAD protein was overexpressed in 58% of our OC cohort. Furthermore, its cytoplasmic overexpression was significantly correlated with tumor grade (*p*= 0.05), tumor subtype (*p*= 0.05), tumor necrosis (*p*= 0.01), and age at menarche (*p*= 0.002). Interestingly, Kaplan-Meier analysis showed a significant correlation of disease-free survival (DFS) with OC patients with cytoplasmic N-CAD overexpression (*p*< 0.03, log rank). Patients with high N-CAD expression have approximately twice the recurrence rate at 5-year follow-up. The results of this study demonstrate a poor prognostic role of N-CAD overexpression in OC, which is reflected in higher recurrence and death rates of OC and its molecular contribution to EMT and distant metastasis. Therefore, OC patients with overexpressed N-CAD need to be monitored more frequently and closely. Further studies with larger patient cohorts are needed to validate these findings, demystify the role of N-CAD in OC pathophysiology, and further investigate its role as a potential therapeutic target.

## Introduction

Ovarian cancer (OC) is the 7^th^ most common cancer in women and the 3^rd^ deadliest gynaecological cancer worldwide (1). In Saudi Arabia, OC affects more than 3% of Saudi women (2–4). This higher mortality rate of OC worldwide seems to be related to the fact that this malignant disease is asymptomatic, especially at early stages (5). In addition, most OC symptoms are nonspecific, misleading and may be confused with other gastrointestinal, urologic, or other diseases (6). Pelvic or abdominal pain and abdominal distension, increased urinary frequency, and some eating disorders such as early satiety are the common OC symptoms in the early stages, while women with advanced stages have a pelvic mass that extends beyond the adnexa (7). OC is classified according to the cellular origin of the malignancy, i.e., epithelial, stromal, or germinal cells. Of note, the vast majority (90%) of OC is of epithelial origin (8). Standard treatment options for OC depend on the type and stage of OC and include surgery along with platinum-based chemotherapy such as carboplatin and paclitaxel, either adjuvant, neoadjuvant, or sometimes both (9). Although 80% of patients diagnosed at an early stage respond to first-line chemotherapy, efficient early diagnosis of OC is still unattainable.

Since most OC patients are not diagnosed until the stage of metastasis, treatment options are not effective enough and are more diverted towards to alleviating symptoms rather than curing the disease. In fact, the 5-year survival rate for OC patients diagnosed with advanced stage disease is about 30% compared to 93% for early stage counterparts (10). Additionally, most OC patients relapse after completion of first-line treatment and require retreatment, mainly with chemotherapy (11). Despite, standard therapies are widely used in the treatment of OC, the prognosis and survival of OC are still poor. In addition, current management and treatment options are challenged by OC heterogeneity, in which a cluster of multiple cells with different genetic and epigenetic features occurs in the same ovarian malignant mass. Furthermore, individuals at the same stage of OC and treated with the same treatment plan have different outcomes. Taken together, these findings highlight the current challenges in optimizing/personalizing current therapeutic strategies for better outcomes (12, 13) and underscore the urgent need for additional effective biomarkers for earlier detection, better prognosis, and more accurate stratification of patients to achieve better individualized treatment options and survival outcomes.

Carbohydrate antigen 125 (CA125) is the first biomarker discovered for the detection of OC. Its level in serum is elevated in most epithelial OC (14). However, the sensitivity of CA125 in OC early stages remains too low, and its level correlates with other diseases such as endometriosis, pregnancy, ovarian cysts, and inflammatory peritoneal diseases. To improve the specificity of OC detection, other biomarkers such as Human Epididymis Protein 4 (HE4) have been developed. HE4 is more sensitive than CA125 and is found in approximately 100% of serous and endometroid subtypes, but its concentration can be influenced by many factors such as body mass index (BMI) (15), smoking (16), and lower HE4 concentration in patients using oral contraceptives (17). Although the combination of CA125 and HE4 has been shown to provide better diagnostic efficacy for risk prediction of OC (18), they are still not accurate and effective enough. More molecular biomarkers are needed to achieve better prognostic, therapeutic and prediction results. Cadherins are important transmembrane glycoproteins that are critical for cell-cell adhesion, especially in epithelial tissues. They were first described as single-pass transmembrane glycoproteins involved in cell–cell adhesion, and are now considered important players in cell polarity and tissue morphology (19). They are also thought to play a direct role in carcinogenesis and metastasis in many cancers (20, 21). In some cases of epithelial carcinoma, epithelial cells lose cell-cell adhesion and polarity and develop migratory and invasive behavior. This process, termed epithelial-mesenchymal transition (EMT), is critical for the development of metastases in cancer progression. A fundamental event in EMT is the “cadherin switch”, defined as loss of E-cadherin expression and increased expression of N-cadherin during cancer progression (22, 23). N-cadherin, also known as CDH2, is a cell-adhesion molecule mapped to 18q11.2 (24). It is a 135 KDa protein that belongs to the family of transmembrane molecules and mediates calcium- dependent intercellular adhesion. It consists of five extracellular cadherin repeats. The cytoplasmic domain of N-cadherin is anchored to the intercellular actin cytoskeleton by interaction with the β-, α-, and γ-catenin complex. *CDH2* is expressed in various tissues, including the nervous system, brain, cardiac and skeletal muscles, blood vessels, and hematopoietic function (25, 26). N-cadherin is mainly expressed in the nervous system and promotes intercellular adhesion of neuronal cells, while its expression is low in normal tissues (25–29). However, it has been reported that overexpressed N-cadherin is associated with cell migration, angiogenesis, aggressiveness, and metastasis in many cancers such as breast, lung, bladder, prostate, and hepatocellular carcinomas (25–29). Moreover, the level of soluble N-cadherin in the serum of cancer patients is much higher than that in healthy individuals. As a result, N-cadherin has been suggested as a potential therapeutic target for tumour invasion and metastasis (30). In OC, the role of N-cadherin expression is unclear and there are few studies that have investigated N-cadherin expression in OC (31), especially in the Arabian peninsula. With this background, this study aimed to evaluate N-CAD protein expression patterns as a potential pro-metastatic molecular biomarker that could help improve OC prognosis and management. The associations between N-CAD protein expression patterns with patients’ clinicopathological parameters and its prognostic value in OC were investigated.

## Patients and Methods

### Patients

Formalin-fixed, paraffin-embedded (FFPE) tissues from patients diagnosed with OC and treated mainly at the departments of pathology and gynaecology at King Abdulaziz University Hospital (KAUH) between 1995 and 2014 were used for this study after obtaining informed consent. This retrospective study includes 117 primary OC patients classified based on histopathological features, mainly according to Tumor Node Metastasis (TNM) classification system. Patients’ medical records were used to collect all pathological and clinical data after IRB approval from KAUH (IRB number: KAUH-189-14).

### Tissue Microarray and Automated Immunostaining

Our group had previously transferred the OC FFPE tissue samples into a tissue microarray (TMA) format. Haematoxylin and Eosin (H&E) from each block (donor block) were used to determine tumor regions. Subsequently, all H&E stained slides from all blocks were reviewed by a pathologist to select the tumour areas to be punched/cored. The details of TMA construction mapping, and validation have been described elsewhere (32, 33).

Immunohistochemistry (IHC) was performed on ovarian cancer TMA slides using an automated staining system (Benchmark XT, Ventana Medical System, Inc. Tucson, Arizona, USA), except for antibodies, which they were applied manually. Reagents were removed from the refrigerator to reach room temperature before starting the run. The slides were labelled with a barcode. The concentrated N-CAD rabbit polyclonal antibody (catalog # ab66025, Abcam, dilution: 1:20) was used. The detailed protocol of the IHC procedure was performed as described elsewhere (32, 34). Briefly, the automated Ventana began the run by deparaffinizing the paraffin-embedded tissue sections with EZ Prep™. They were then pre-treated with Cell Conditioning buffer (CC1) to induce/activate the epitopes of the antigens (antigen retrieval). Then, 50μl of the optimized antibody was applied manually for 30 minutes at room temperature. This was followed by washing steps using the UltraView Universal DAB Detection Kit (Lot. No. E00534) which, contains: Copper, 1.1% hydrogen peroxide solutions, DAB substrate, SA-HRP contains a conjugated streptavidin horseradish peroxidase solution and inhibitor. For counterstaining, staining was completed with hematoxylin II for 8 min. and post-counterstaining by bluing reagent for 4 min.

After completion of the run, the slides were removed from the instrument and rinsed with a mild detergent followed by tap water to remove LCS and buffer residue. Then the slides were immersed in different concentrations of alcohol buffer (70, 95 and 100%) and then cleaned in xylene, for 3 minutes, twice for each solution. Finally, a drop of mounting medium was added to the slide and covered with a glass coverslip. The stained slides were manually scored to check the expression of the biomarkers under the light microscope using the staining patterns.

### Scoring and Evaluation of Biomarkers Expression

Evaluation of protein expression of all OC was assessed using a regular Nikon light microscope at ×40 magnification blind to the clinicopathological parameters of the patients. The staining was classified into four groups: 1) negative 2) weak 3) moderate and 4) high expression. The intensity of staining and the percentage of positively stained cells were used to calculate the staining index score according to the following formula


I= 0xf0 + 1xf1 + 2xf2 + 3xf3

Where (I) is the staining index score and (f0 to f3) are the proportions of cells that have a given staining intensity (from 0 to +3) (33, 35). This I score is useful for the selection of the best IHC expression cut-off/discriminator during statistical analysis.

### Statistical Analysis

Statistical analyses were performed using the SPSS^®^ software package (version 22). The frequency tables were analyzed using the chi-square test to assess the significance of the correlation between the categorical variables (age, stage, grade, BMI, lymph node status, recurrence, …).

Univariate survival analysis was performed using the Kaplan-Meier method. Tests with *p* < 0.05 were considered statistically significant.

## Results

### Expression Pattern of N-Cadherin Protein Profiles in Ovarian Cancer

**Figure 1:**
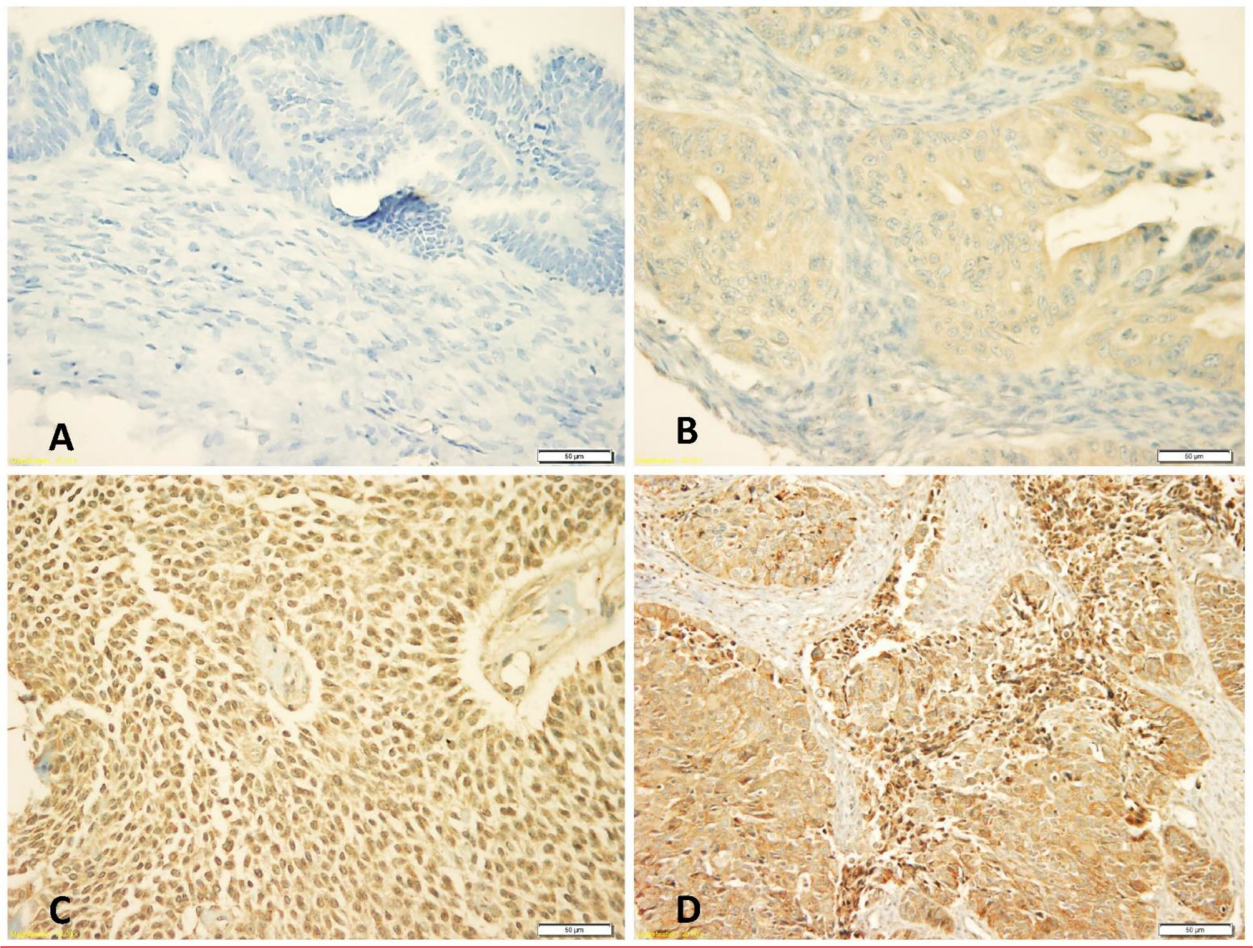
Immunohistochemical staining patterns of cytoplasmic N-cadherin protein expression at 40x magnification: **(A)** Negative cytoplasmic expression, **(B)** Weak cytoplasmic expression, **(C)** Moderate cytoplasmic expression, **(D)** Strong cytoplasmic expression.

Expression of N-cadherin protein was observed in both membrane and cytoplasm, but mainly in cytoplasm. The frequencies of expression patterns of cytoplasmic N-cadherin protein receptors in 117 OC samples evaluated by the IHC technique were: no expression (0, 3%), weak expression (+1, 39%), moderate expression (+2, 44%) and strong expression patterns (+3, 14%), respectively (**Figure 1**).

### Correlation Of Cytoplasmic N-Cadherin Protein Expression With Clinicopathological Features

Our data showed that cytoplasmic N-cadherin expression was not associated with age, lymph node involvement, and tumor stage. However, significant correlations were found with tumor grade, tumor subtype, tumor necrosis, and age at menarche. In poorly differentiated tumors, expression of N-Cad was low compared to well/intermediately differentiated tumors (*p*= 0.05). Among histological subtypes, serous tumors showed low N-CAD expression compared to mucinous/other subtypes that showed high expression of N-CAD profile (*p*= 0.05). On the other hand, OC tissues with tumor necrosis showed high N-cad expression compared to their counterparts (*p*= 0.01). Interestingly, OC patients with early onset of menarche had tumors with high N-cad expression (*p*= 0.002) (**Table 1**).

**Table 1:** Correlation between cytoplasmic N-cadherin protein expression patterns and clinicopathological features of OC.

Patients features	Number of cases (%)	Cytoplasmic N-cadherin Protein Expression patterns: N (%)		*p*-value
		Low Expression (0, 1+)	High Expression (2+, 3+)	
Age				
< 50	67 (57%)	26 (39%)	41 (61%)	0.62
> 50	49 (42%)	24 (49%)	25 (51%)
Missing	1 (1%)		
Tumor size				
1-5 cm	25 (21%)	12(48%)	13 (52%)	0.90
6-10 cm	30 (26%)	13 (43%)	17 (57%)
>10 cm	57 (49%)	23 (41%)	34 (59%)
Missing	5 (4%)		
Histological subtype				
Serous	50 (43%)	26 (52%)	24 (48%)	0.05
Mucinous	28 (24%)	9 (32%)	19 (68%)
Other types	35 (30%)	12 (34%)	23 (66%)
Missing	4 (3%)		
Tumor grade				
low grade (WD)	15 (13%)	7 (47%)	8 (53%)	0.05
Intermediate	19 (16%)	5 (26%)	14 (74%)
High grade (PD)	63 (54%)	33 (52%)	30 (48%)
Missing	20 (17%)		
Lympho-vascular invasion				
Negative	54 (46%)	23 (43%)	31 (57%)	0.35
Positive	39 (33%)	18 (46%)	21 (54%)
Missing	24 (21%)		
Tumor necrosis				
Negative	57 (49%)	18 (31%)	39 (69%)	0.01
Positive	45 (38%)	25 (56%)	20 (44%)
Missing	15 (13%)		
BMI				
< 23	8 (7%)	1 (13%)	7 (87%)	0.43
23-26	28 (24%)	10 (36%)	18 (64%)
> 26	52 (44%)	25 (48%)	27 (52%)
Missing	29 (25%)		
Age of menarche				
< 13	19 (16%)	1 (5%)	18 (95%)	0.002
> 13	67 (57%)	36 (54%)	31 (46%)
Missing	31 (27%)		
Tumor stage				
Low stage (I,II)	41 (35%)	19 (46%)	22 (54%)	0.77
High stage (III,IV)	66 (56%)	28 (43%)	38 (57%)
Missing	10 (9%)		
Recurrence status				
None	51 (44%)	21 (41%)	30 (59%)	0.71
Yes	36 (31%)	14 (39%)	22 (61%)
Missing	30 (25%)		

Red p-values are statistically significant.

### Correlation Of Cytoplasmic N-Cadherin Protein Expression With Survival Outcome

**Figure 2:**
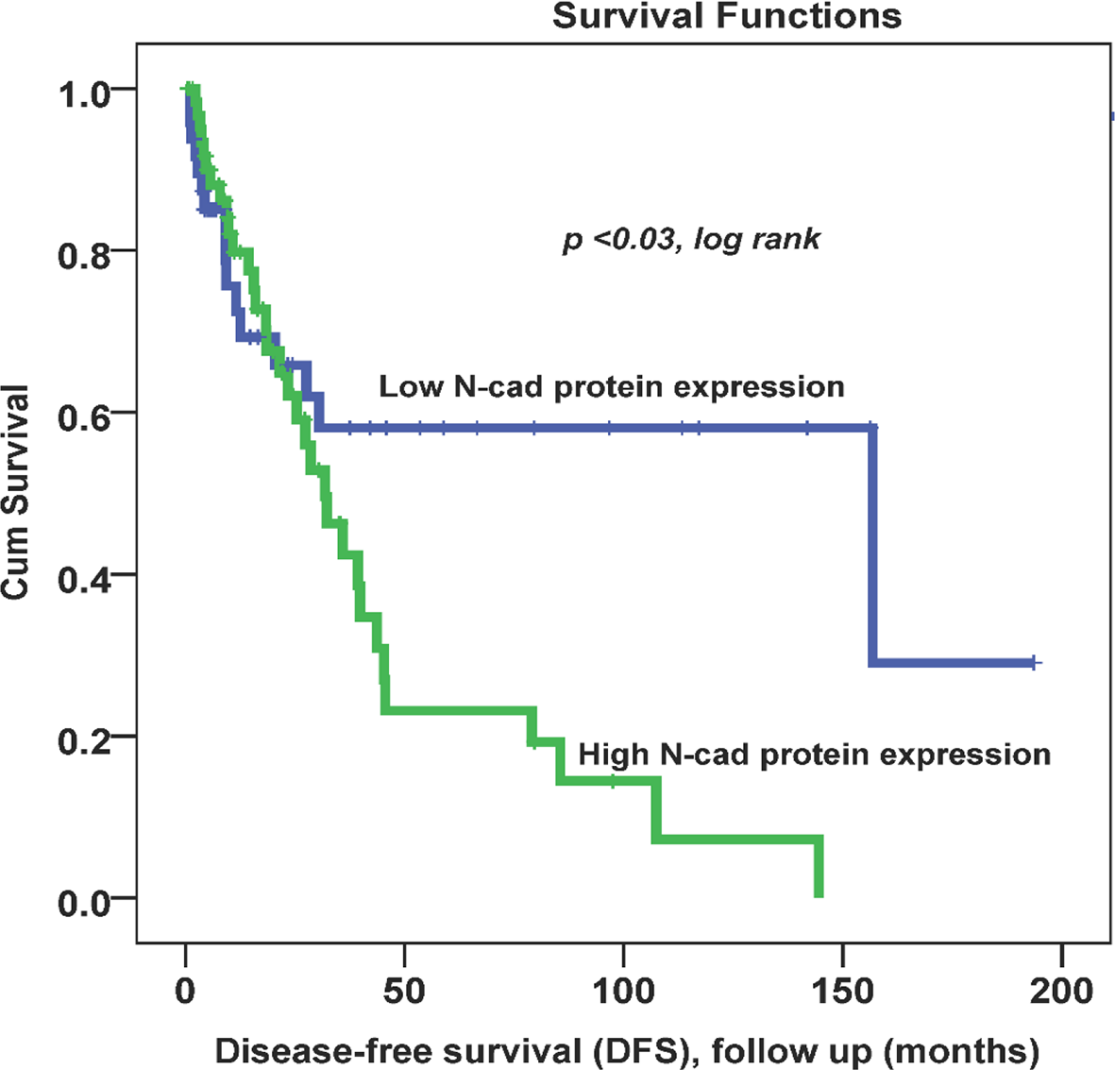
Cytoplasmic N-cadherin expression patterns in OC cohort using the cut-off (low (0, 1+) vs. high (2+, 3+)) as a determinant of disease-free survival (DFS) in univariate (Kaplan-Meier) analysis (*p* < 0.03, log-rank).

**Figure 3:**
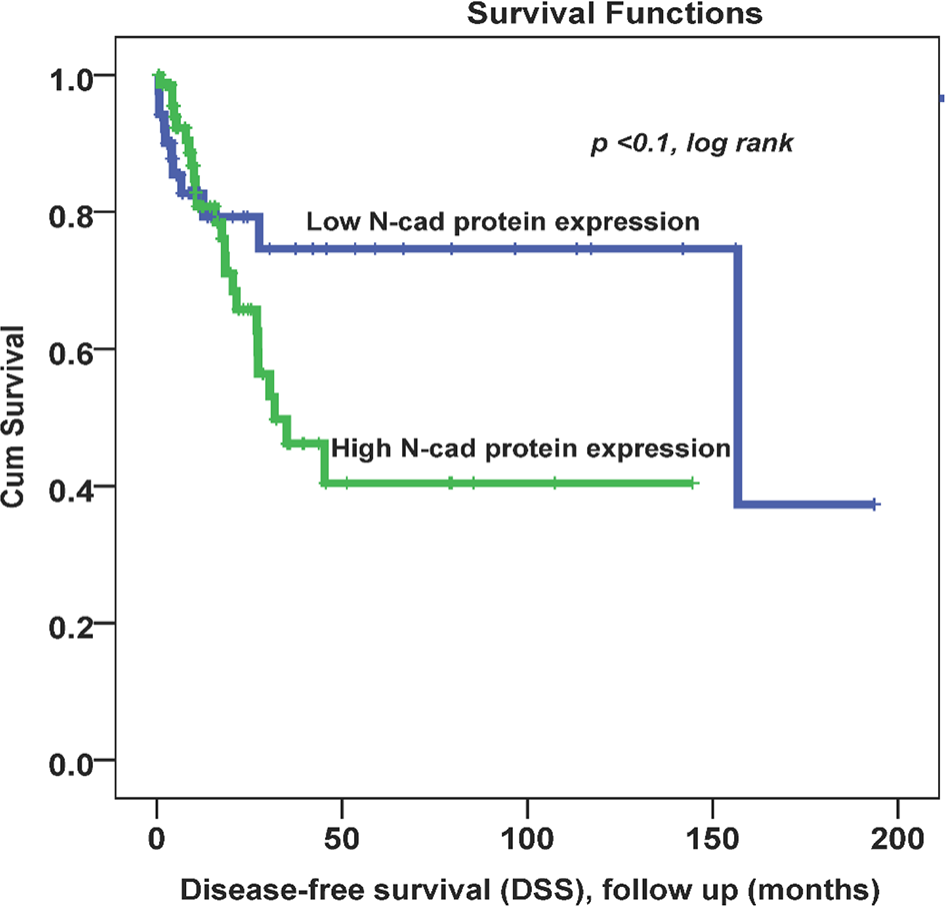
Cytoplasmic N-cadherin expression patterns in OC cohort using the cut-off (low (0, 1+) vs. high (2+, 3+)) as a determinant of disease -specific survival (DSS) in univariate (Kaplan-Meier) analysis (*p* < 0.1, log-rank).

Throughout the follow-up period, univariate survival analyses with a cut-off point for N-cad expression (low (0, 1+) vs. high expression (2+,3+)) as a discriminator showed the best prognosis. Thus, at 5 years, disease recurrence occurred in 42% of patients whose OC tissues had low N-cad expression compared with approximately 78% of patients whose OC tissues had high N-cad protein expression (*p* < 0.03, log rank, **Figure 2**). On the other hand, the same trend was observed with less significance in patients who died from the disease. Using the same cut-off point described above, approximately 22% of patients whose OC tissues had low N-cad expression died compared to approximately 60% of patients who had high N-CAD expression in their OC tissues (*p*=0.1, log rank, **Figure 3**). The Kaplan-Meier survival curves clearly show that shorter survival was associated with high N-cad protein expression, while patients with low N-cad expression had a lower recurrence rate and thus longer survival.

## Discussions

In 2021, more than 21,000 new cases were diagnosed with OC worldwide, and about 13,770 patients have died from this deadly disease (36). Several factors are believed to contribute to this increasing incidence and higher mortality rates. Apart from the difficulty of detecting the disease at early stages, the OC treatment options are not very effective at advanced stages, mainly because of the heterogeneity and complexity of this malignant disease. The higher complexity of OC is the result of various intertwined genetic and epigenetic factors that lead to aberrant gene expression and inconsistent treatment outcomes (37). In conjunction with clinicopathological factors such as age, grade, stage, and lymph node invasion, OMICs tools have provided an unprecedented understanding of the molecular complexity and disease progression of the diseases. In particular for OC, many genes have been reported as mutated, including *BRCA1, BRCA2, BRIP1, RAD51C, RAD51D, MSH2, MLH1, PMS2*, and *MSH6*. These and other candidate genes have been associated with a higher risk of OC (38, 39). Despite numerous efforts to identify reliable OC biomarkers, early detection strategies still rely mainly on CA125 and HE4, which have not been shown to be specific and sensitive enough (40, 41). Therefore, additional efforts are needed to develop new theranostic tools that can alleviate the suffering of OC patients and improve the treatment of the disease. Currently, the focus is on identifying more effective and clinically useful prognostic markers at the genomic and proteomic levels to detect OC at an early, curable stage and potentially support therapeutic decision making. In this regard, N-CAD has been reported to be expressed in several cancer types and has been associated with several clinicopathological parameters as well as survival outcomes. However, the clinical and prognostic significance of N-CAD in OC has not been well studied, especially in the Arabic Peninsula. Therefore, we conducted this study to investigate the N-CAD expression patterns and evaluate its prognostic value in our cohort of OC patients.

Our study showed that the protein N-CAD was expressed mainly in the cytoplasm of 58% of our patients’ tumor cells, with a recorded expression also in the cell membrane. Similarly, Quattrocchi et al. reported that 99% (158 cases) of their OC cohort expressed N-CAD protein in the cytoplasm (42). However, other studies reported membranous N-CAD expression in 32% of their cohort (43). These discrepancies could be due to cohort size, ethnicity, proportion of histological subtypes, and the complicated molecular heterogeneity of OC within each subtype (44).

The results of this study showed also that the expression of N-CAD protein was significantly associated with some clinicopathological characteristics including histological subtype, grade, tumor necrosis and age of menarche (*p*< 0.05) (**Table 1**). These findings are consistent with many studies that reported significant correlations of N-CAD expression in OC with histologic subtypes (45, 46) and grade (45). On the other hand, they found a significant association between N-CAD protein expression and tumor stage, which is not confirmed by our results. Furthermore, and in agreement with our results, other studies on OC reported no significant correlation between the expression of N-CAD and other clinicopathological parameters such as tumor stage, patient’s age, BMI, and tumor size (43, 47). Our results showed that 57% of our patients’ cohort were below 50 years (**Table 1**). There is a noticeable early onset of OC in the Saudi population compared to the United Kingdom for example where, according to Cancer Research UK, 53% of OC cases were diagnosed at 65 and over. Possible reasons associated to genomic, environmental and lifestyle factors deserve to be investigated to explain this early onset phenomenon.

In Kaplan-Meier survival analysis, N-CAD protein expression was significantly associated with DFS (*p*=0.03). In fact, patients with higher N-CAD expression have approximately twice the recurrence rate at 5-year follow-up time (42% vs. 78% recurrence at 60 months; p < 0.03, log rank, **Figure 2**). A similar trend was also observed with lower significance for DSS, in which patients with higher N-CAD expression who died more rapidly from the disease compared with their counterparts with low N-CAD expression (**Figure 3**). In general, the Kaplan-Meier survival curves clearly show that shorter survival and higher recurrence rates were associated with overexpression of the N-CAD protein. These results are consistent with those of Quattrocchi *et al.*, who reported that all patients in their cohort with N-CAD overexpression relapsed by the first year of follow-up time. In the same study, patients with higher E-CAD expression survived shorter than their counterparts with lower N-CAD expression (42). Two important meta-analysis studies using all published data and freely available sources about N-Cadherin showed similar survival outcomes as our results. In fact, they confirmed that N-CAD overexpression is a negative prognosticator of OC (48, 49). When we assessed the freely available KM plotter analysis of TCGA database (https://kmplot.com/analysis/), it appears that this platform did not cover the N-Cadherin protein expression (CDH2) in OC. However, the mRNA data showed that *CDH2* gene expression is a good prognosticator of OC (50) (http://kmplot.com/analysis/index.php?p=service). For the protein Atlas database (https://www.proteinatlas.org), the CDH2 protein was not a significant prognosticator in OC possibly due to the heterogeneity of the cohort (51) (https://www.proteinatlas.org/ENSG00000170558-CDH2/pathology).

The survival data indicated that poor disease progression associated with high N-CAD protein expression appears to be either a marker of OC aggressiveness or actively involved in the pathophysiology of disease progression, recurrence, and metastasis. Similar studies in other cancers (breast, lung, bladder, prostate, …) confirmed that overexpression of N-CAD protein was associated with poor treatment outcomes, cell migration, angiogenesis, disease aggressiveness, and metastasis (25–29). Thus, overexpression of N-cadherin in colorectal cancer was significantly associated with poor disease-specific survival and disease-free survival, as well as with many clinicopathological characteristics such as tumor size, lymph node, stage, and grade (52). Similarly, high expression of N-cadherin in bladder cancer was shown to be associated with grade, tumor stage, and poorer recurrence- free survival (53).

**Figure 4:**
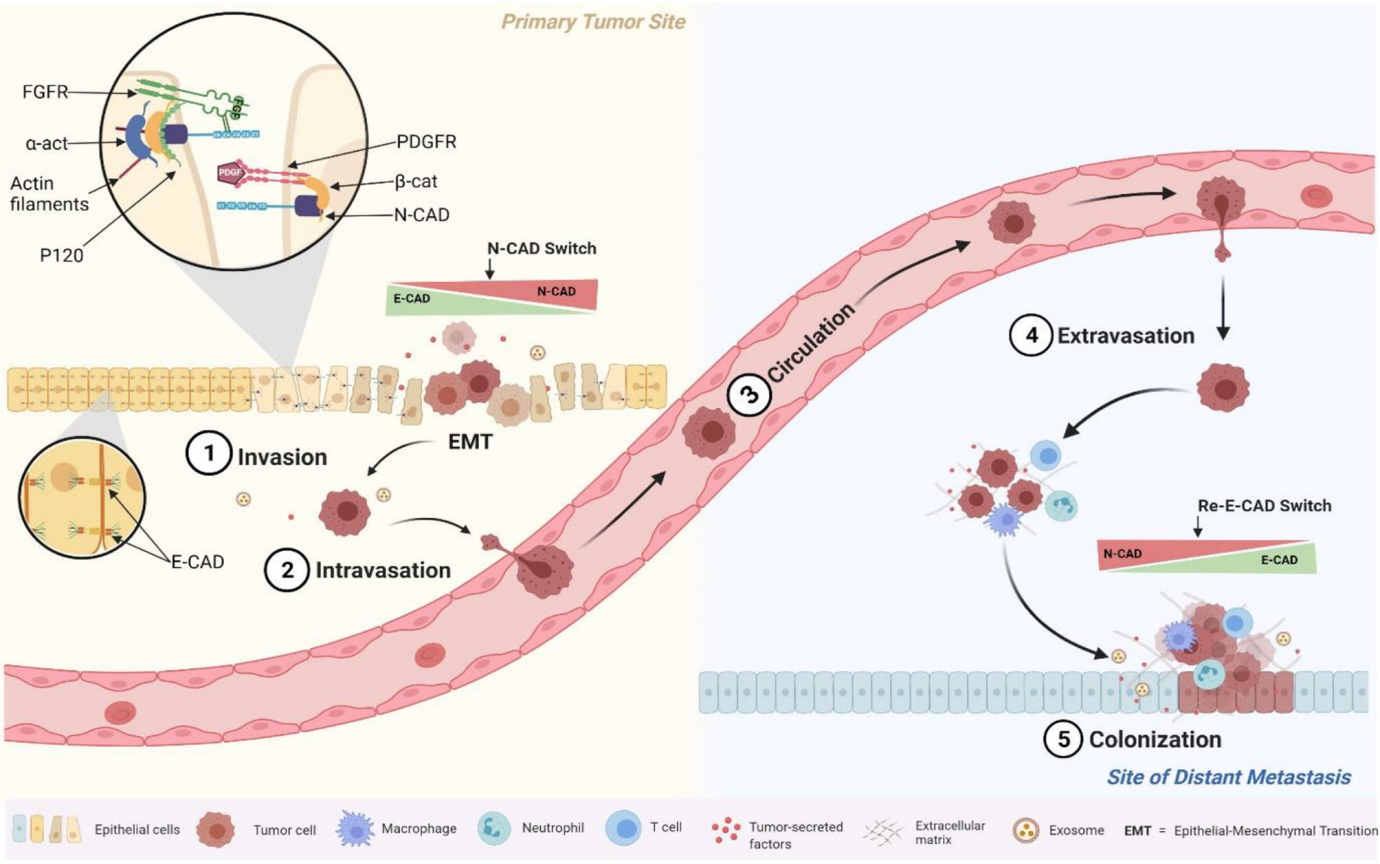
Schematic illustration of the molecular and cellular events triggered by the N-cadherin switch at EMT. Overexpressed N-cadherin binds and stabilizes FGFR on the cell surface to initiate cell signalling cascades, and to PDGFR to increase cell motility through interactions with ß-catenin.

Taken together, these results seem to be related to the role of N-CAD in the mesenchymal phenotype, which promotes cell mobility and invasion (31, 54). In fact, several reports have shown that when epithelial tumor cells switch from expressing E-CAD to expressing N-CAD (cadherin switch phenomenon), they acquire the ability to activate Fibroblast Growth Factor Receptor (FGFR) pathways. Our results together with our previous study about E-Cad expression in the same cohort confirmed the cadherin switch (32). In fact, while the E-CAD expression was decreasing at the advanced stages (starting from the EMT and marked by cancer invasion and migration), the N-CAD expression was increasing; and both markers were prognosticators of poor survival outcomes (32). In fact, once N-CAD is overexpressed, it has been shown to affect tumor cell polarity and behavior through its direct interaction with the FGFR, which regulates cancer cell motility and invasion (55, 56). Also, N-CAD was reported to interact with other receptors on tumor cells to promote motility and migration such as Platelet Derived Growth Factor Receptor (PDGFR). This mechanism occurs when the NHERF protein binds the N-CAD with the ß-catenin to the PDGFR to form a complex that drives tumor cells to migrate and motility (56) (**Figure 4**). These molecular mechanisms of N- CAD protein overexpression, summarized in **Figure 4**, played a key role in the phenotypic changes of tumor cells that were actively involved in migration to distant metastases. This pro-metastatic role of N-CAD was also confirmed *in vitro* with epithelial cells engineered to overexpress N-CAD. These cells have been shown to alter their morphology and behavior, adopting a motile phenotype similar to that observed in cells undergoing EMT (57, 58). This pro-metastatic phenotype depends also on the expression of other interacting proteins in addition to N- CAD, as mentioned previously and summarized in **Figure 4** (58, 59).

This study demonstrated a prognostic role of N-CAD in OC, the first to be reported in the Arabic Peninsula. OC patients overexpressing the N-CAD protein had a poor prognosis, as evidenced by higher rates of both OC recurrence and death, as well as its molecular contribution in EMT and distant metastasis; and thus required more frequent and closer follow-up. Further studies with larger patient cohorts are needed to validate these findings, investigate further the role of N-CAD in OC pathophysiology, and explore its role as a potential therapeutic target.

## Data Availability Statement

The original contributions presented in the study are included in the article/supplementary material. Further inquiries can be directed to the corresponding author.

## Ethics Statement

The study was approved by the Institutional Ethical Review Board of King Abdulaziz University Hospital, Jeddah, Saudi Arabia (Ref. number: KAUH-189-14). The patients/participants provided their written informed consent to participate in this study.

## Author Contributions

The author confirms being the sole contributor of this work and has approved it for publication.

## Funding

This work was funded by the Deanship of Scientific Research (DSR), King Abdulaziz University, Jeddah, under grant No. (D-545-117-1443). The author, therefore, gratefully acknowledges the DSR technical and financial support.

## Conflict of Interest

The author declares that the research was conducted in the absence of any commercial or financial relationships that could be construed as a potential conflict of interest.

## Publisher’s Note

All claims expressed in this article are solely those of the authors and do not necessarily represent those of their affiliated organizations, or those of the publisher, the editors and the reviewers. Any product that may be evaluated in this article, or claim that may be made by its manufacturer, is not guaranteed or endorsed by the publisher.
